# Factors associated with knowledge, attitude and practice related to hepatitis B and C among international students of Universiti Putra Malaysia

**DOI:** 10.1186/s12889-016-3188-5

**Published:** 2016-07-21

**Authors:** Abdulrahman Ahmad, Lye Munn Sann, Hejar Abdul Rahman

**Affiliations:** Department of Community Health, Faculty of Medicine, Universiti Putra Malaysia, Serdang, Selangor Malaysia; Department of Medicine, Specialist Hospital Sokoto, Sokoto, Nigeria

**Keywords:** Knowledge, Attitude, Practice, Hepatitis B, Hepatitis C, International students

## Abstract

**Background:**

Knowledge of hepatitis B and C has been reported to be low among respondents in different studies. We conducted a cross-sectional study among international students of Universiti Putra Malaysia (UPM) to ascertain their levels of knowledge, attitude and practices regarding hepatitis B and C and its associated factors.

**Methods:**

Six hundred and sixty two (662) international students participated in this study. A cluster sampling method was employed and data was generated using self-administered questionnaire, which was validated and its reliability checked.

**Results:**

Normality test was conducted followed by descriptive statistics, spearman’s correlation and Chi-square tests to explore associations between variables in the study. The response rate was 71.49 %. Of these, 50.3 % of the respondents had better knowledge of hepatitis B; 52.7 % had better knowledge of hepatitis C; 54.8 % had positive attitude towards hepatitis B and C and 77.6 % had safer practices towards hepatitis B and C. Positive correlations were found between knowledge of hepatitis B and knowledge of hepatitis C; knowledge hepatitis B and attitude; knowledge hepatitis C and attitude; knowledge hepatitis B and practice; knowledge hepatitis C and practice; and attitude and practice regarding hepatitis B and C. Similarly, some socio-demographic variables and history of hepatitis were found to be associated with knowledge, attitude and practice related to hepatitis B and C.

**Conclusion:**

The levels of knowledge and attitude towards hepatitis B and C were low among respondents but majority of them exhibited safe practices. The study level, faculty, age, nationality, marital status and gender of the respondents were significantly associated with their levels of knowledge, attitude and practices towards the disease. These findings imply that there is need for hepatitis health promotion among the international students of UPM and possibly other international students across the globe. It will serve to improve their levels of knowledge, attitude and practices in short term and get them protected against the disease in the long run.

## Background

Knowledge of hepatitis is shown to be low among different populations [1–6]. It is estimated that one out of every twelve people in the world is chronically infected with either hepatitis B or C. These result in about 1 million deaths annually. Hepatitis B and C are also responsible for about 78 % of all primary liver cell carcinoma and 57 % of all cases of liver cirrhosis. About 2 billion people have been infected with hepatitis B globally, resulting in 350 million hepatitis B virus carriers globally and 600,000 deaths annually. It is also estimated that the global prevalence (hepatitis B surface antigen positive) of hepatitis B infection is 5 % but ranges between 0.1 to 20 % among the low and high endemic areas [7, 8].

Globally, there are about 130–170 million carriers of hepatitis C virus, this results in about 350,000 of global mortality annually. Prevalence (antibody seropositive) of hepatitis C globally stands at 3 % [8, 9], but it ranges between 0.2 to 18 % depending on the geographical region [9, 10]. Malaysia experienced relative drop in prevalence of hepatitis B from 5–7 % before 1989 to between 0.4 to 2.5 % depending on the region in 1996 and now to 0.62 % [11] among young age group entering university. Malaysia is considered to be among the low endemic countries as the prevalence of hepatitis B has declined to below 2 %. The prevalence of hepatitis C in the country was estimated to be around 1.5 % [12, 13]. The trend can be reversed with increase in international students coming into Malaysia if they are not aware of the risk factors of the disease and they became infected along their study period. This is because some of the students came from hepatitis endemic countries like China and Indonesia among other countries. This is more so with regards to hepatitis C which has no vaccine and is believed to be more common among intravenous drug abusers, which is a factor in Malaysia.

This study aimed to assess the levels of knowledge, attitude and practice related to hepatitis B and C among international students of UPM and the associated factors (socio-demographic factors and past history related to hepatitis B and C) to give room for intervention study if the levels were found to be low.

## Methods

### Design

The data for this cross-sectional study was collected between 16^th^ April, 2014 and 28^th^ May, 2014 among international students of UPM at Serdang campus. Cluster sampling method was used with university’s faculties taken as clustering units. Samples were selected randomly using table of random numbers. Seven (7) out of 15 faculties of the university were randomly selected based on calculation of the minimum require clusters needed from the sample size and average number of students per cluster (faculty). Efforts were made to design the study such that the respondents are representative of the study population as much as possible. This was achieved by employing probability sampling and reaching out to reasonable number of respondents in a short period of time to avoid any contamination. Participation in the study was completely voluntary and anonymous and no incentive was given to the respondents. Respondents were recruited in their lecture hall (before the lectures begin), respective laboratories or hostels.

The international students of UPM came from all continents of the world, but majority are from the Asian continent. Both undergraduates and postgraduates were included in the study. Inclusion criteria require being an active and registered international student of UPM at Serdang Campus. The international students that were not available during data collection were excluded from the study.

Sample size was estimated based on the finding of level of knowledge regarding hepatitis B and C among university students in Pakistan. It was found to be 45.9 and 31 % for those aged 20–21 and those aged greater than 21 respectively [14]. Therefore, formula for 2 proportions was used [15]. The estimated sample size was 742.

### Instrument

A questionnaire, which was self-administered and written in English was used as instrument for data collection in this study. Content and face validities as well as reliability tests were conducted on the questionnaire before it was used for the study. The questionnaire had 63 items grouped into five sections: socio-demographic, history of hepatitis, knowledge, attitude and practice sections.

### Statistical analysis

SPSS version 21 was used for data analysis and *P*-value of equal to or less than 0.05 was taken as significant.

Descriptive analysis was initially conducted, which was then followed by normality test. The data was not normally distributed as such the following statistical tests were conducted:

1. Chi-square test was conducted to determine the association of independent variables with the outcome variable of interest (knowledge, attitude and practice related to hepatitis B and C).
2. Spearman’s correlation was used to determine the correlation between knowledge hepatitis B, knowledge hepatitis C, attitude and practice.

## Results

### Response rate and normality test

The successfully completed and returned questionnaires were 662 and eligible respondents were 926, giving an overall response rate of 71.49 %. About 192 international students were not available during data collection. Most of them went to their respective countries for data collection or for personal reasons as such they were excluded from the study. Normality test was conducted and the data was not normally distributed with Kolmogorov-Smirnov and Shapiro-Wilk tests showing *p*-value of less than 0.001.

### Distribution of the respondents by socio-demography and history of hepatitis

Table 1 shows the distribution of respondents according to their socio-demographic characteristics. A total of 662 international students participated in the study out of which 388 (58.6 %) were males, and 274 (41.4 %) were females. The ages of the respondents ranged between 18 and 56 years, with median age of 26 years (inter quartile range = 26–37). Two hundred and twenty four respondents did not report their monthly income. However, the monthly income of respondents who reported theirs ranged between (in Malaysian Ringgit (RM)) RM 100–RM 16,000 with mean of RM 2545.16.

**Table 1:** Distribution of respondents by socio-demographic characteristics (*N* = 662)

Socio-demographic characteristics	f	%
Faculty		
Agriculture	105	15.9
Economics and Management	135	20.4
Educational studied	72	10.9
Human Ecology	49	7.4
Modern Languages and Communication	124	18.7
Sciences	104	15.7
Veterinary Medicine	73	11.0
Gender		
Male	388	58.6
Female	274	41.4
Age group (years)		
≤ 20	36	5.4
21–29	220	33.3
≥ 30	406	61.3
Nationality		
Africans	231	34.9
Asians	427	64.5
Europeans	4	0.6
Income (RM)		
≤ 1000	82	12.4
1001–2999	215	32.5
≥ 3000	141	21.3
Not specified	224	33.8
Study level		
Undergraduate	120	18.1
Masters	206	31.1
PhD	336	50.8
Marital status		
Single	318	48.0
Married	339	51.2
Divorced	5	0.8
Widow/widower	0	0

Table 2 shows the distribution of the respondents according to past history related to hepatitis. From the results as shown in the table above only 38.1 % of the respondents were vaccinated.

**Table 2:** Distribution of respondents by history of hepatitis (662)

History of hepatitis	f	%
Family		
Yes	45	6.8
No	617	93.2
Acquaintance		
Yes	197	29.8
No	464	70.1
Yellowness of the eyes		
Yes	41	6.2
No	621	93.8
Personal		
Yes	27	4.1
No	635	95.9
Hepatitis B vaccination status		
Yes	252	38.1
No	410	61.9
Doses of vaccine receive		
0	311	47.0
1	31	4.7
2	42	6.3
3	95	14.4
Do not know	183	27.6

### Levels of knowledge attitude and practice

The findings revealed that the percentages of international students in UPM with better knowledge of hepatitis B and C (Better knowledge is achieved by the respondent when the respondent obtained aggregate knowledge score of median (8 for hepatitis B and 6 for hepatitis C with inter quartile ranges of 5–10 and 2–9 respectively) or above and less knowledge for those with aggregate scores less than median score) are 50.3 % and 52.7 % for hepatitis B and hepatitis C respectively. The prevalence of positive attitude (Positive attitude is considered when a respondent got aggregate of median attitude score (25 with inter quartile range of 22–28) or above and negative attitude for those with less than aggregate scores less than median) among the respondents is 54.8 %, indicating that more than half of the respondents have positive attitude towards the disease. Similarly, the proportion of participants with safer practices (Safe practice is when a respondent got aggregate practice scores of median (6 with inter quartile range of 6–7) or above and unsafe practice for those with less than median practice score.) towards hepatitis B and C is shown to be good as 77.6 % of the respondents exhibited safe practices.

### Correlation of knowledge, attitude and practice regarding hepatitis B and C

There were significant positive correlations between combinations of these variables: with strongest correlation found between knowledge of hepatitis B and hepatitis C r 0.73, *p*-value <0.001; knowledge of hepatitis B with practice r 0.154, *p*-value <0.001; knowledge of hepatitis B with attitude r 0.373, *p*-value <0.001; knowledge of hepatitis C with attitude r 0.329, *p*-value <0.001; knowledge of hepatitis C with Practice r 0.141, *p*-value <0.001 and weakest correlation was between attitude and practice towards hepatitis B and C r 0.11, *p*-value 0.004.

### Comparison between levels of knowledge, attitude and practice and socio-demographic factors as well as history of hepatitis

Table 3 shows factors associated with knowledge, attitude and practice related to hepatitis B and C among international students of Universiti Putra Malaysia.

**Table 3:** Factors associated with hepatitis B and C knowledge, attitude and practice (*N* = 662)

Variable		Knowledge HB		Knowledge HC		Attitude		Practice	
		Better f (%)	Poor f (%)	Better f (%)	Poor f (%)	Positive f (%)	Negative f (%)	Safe f (%)	Unsafe f (%)
Gender	Male	191 (49.2)	197 (50.8)	192 (49.5)	196 (50.5)	211 (54.4)	177 (45.6)	288 (74.2)	100 (25.8)
	Female	142 (51.8)	132 (48.2)	157 (57.3)	117 (42.7)	152 (55.5)	122 (44.5)	226 (82.5)	48 (17.5)
		*χ* ^2^ = 0.43	*p* = 0.510	*χ* ^2^ = 3.94	*p* = 0.047	*χ* ^2^ = 0.08	*p* = 0.781	*χ* ^2^ = 6.30	*p* = 0.012
Faculty	Sciences	156 (55.3)	126 (44.7)	160 (56.7)	122 (43.3)	178 (63.1)	104 (36.9)	219 (77.7)	63 (22.3)
	Arts & Humanities	177 (46.6)	203 (53.4)	189 (49.7)	191 (50.3)	185 (48.7)	195 (51.3)	295 (77.6)	85 (22.4)
		*χ* ^2^ = 4.95	*p* = 0.026	*χ* ^2^ = 3.18	*p* = 0.074	*χ* ^2^ = 13.62	*p* < 0.001	*χ* ^2^ = 0.00	*p* = 0.993
Nationality	Africans	117 (50.6)	114 (49.4)	123 (53.3)	108 (46.8)	156 (67.5)	75 (32.5)	185 (80.1)	46 (19.9)
	Asians	213 (49.9)	214 (50.1)	224 (52.5)	203 (47.5)	203 (47.5)	224 (52.5)	326 (76.3)	101 (23.7)
	Europeans	3 (75.0)	1 (25.0)	2 (50.0)	2 (50.0)	4 (100.0)	0 (0.0)	3 (75.0)	1 (25.0)
		*χ* ^2^ = 1.06	*p* = 0.588^a^	*χ* ^2^ = 0.05	*p* = 0.976^a^	*χ* ^2^ = 29.40	*p* < 0.001^a^	*χ* ^2^ = 1.24	*p* = 0.538^a^
Study level	Undergraduates	57 (47.5)	63 (52.5)	61 (50.8)	59 (49.2)	41 (34.2)	79 (65.8)	80 (66.7)	40 (33.3)
	Masters	89 (43.2)	117 (56.8)	98 (47.6)	108 (52.4)	112 (54.4)	94 (45.6)	155 (75.2)	51 (24.8)
	PhD	187 (55.7)	149 (44.3)	190 (56.5)	146 (43.5)	210 (62.5)	126 (37.5)	279 (83.0)	57 (17.0)
		*χ* ^2^ = 8.38	*p* = 0.015	*χ* ^2^ = 4.34	*p* = 0.114	*χ* ^2^ = 28.69	*p* < 0.001	*χ* ^2^ = 14.64	*p* = 0.001
Age group	≤20	19 (52.8)	17 (47.2)	21 (58.3)	15 (41.7)	10 (27.8)	26 (72.2)	22 (61.1)	14 (38.9)
	21–29	109 (49.5)	111 (50.5)	113 (51.4)	107 (48.6)	102 (46.4)	118 (53.6)	168 (76.4)	52 (23.6)
	≥30	205 (50.5)	201 (49.5)	215 (53.0)	191 (47.0)	251 (61.8)	155 (38.2)	324 (79.8)	82 (20.2)
		*χ* ^2^ = 0.15	*p* = 0.930	*χ* ^2^ = 0.63	*p* = 0.731	*χ* ^2^ = 25.02	*p* < 0.001	*χ* ^2^ = 6..96	*p* = 0.031
Marital status	Single	152 (47.8)	166 (52.2)	166 (52.2)	152 (47.8)	158 (49.7)	160 (50.3)	231 (72.6)	87 (27.4)
	Ever married	181 (52.6)	168 (47.4)	183 (53.2)	161 (46.8)	205 (59.6)	139 (40.4)	283 (82.3)	61 (17.7)
		*χ* ^2^ = 1.53	*p* = 0.215	*χ* ^2^ = 0.07	*p* = 0.798	*χ* ^2^ = 6.55	*p* = 0.010	*χ* ^2^ = 8.82	*p* = 0.003
Family history of hepatitis	Yes	31 (68.9)	14 (31.1)	33 (73.3)	12 (26.7)	31 (68.9)	14 (31.1)	37 (82.2)	8 (17.8)
	No	302 (48.9)	315 (51.1)	316 (51.2)	301 (48.8)	332 (53.8)	285 (46.2)	477 (77.3)	140 (22.7)
		*χ* ^2^ = 6.67	*p* = 0.010	*χ* ^2^ = 8.23	*p* = 0.004	*χ* ^2^ = 3.85	*p* = 0.050	*χ* ^2^ = 0.58	*p* = 0.445
Friend with hepatitis	Yes	117 (59.4)	80 (40.6)	121 (61.4)	76 (38.6)	133 (67.5)	64 (32.5)	155 (78.7)	42 (21.3)
	No	216 (46.6)	248 (53.4)	228 (49.1)	236 (50.9)	230 (49.6)	234 (50.4)	359 (77.4)	105 (22.6)
		*χ* ^2^ = 9.12	*p* = 0.003	*χ* ^2^ = 8.37	*p* = 0.004	*χ* ^2^ = 17.98	*p* < 0.001	*χ* ^2^ = 0.14	*p* = 0.711
Yellowness of the eyes	Yes	20 (48.8)	21 (51.2)	23 (56.1)	18 (43.9)	20 (48.8)	21 (51.2)	32 (78.0)	9 (22.0)
	No	313 (50.4)	308 (49.6)	326 (52.5)	295 (47.5)	343 (55.2)	278 (44.8)	482 (77.6)	139 (22.4)
		*χ* ^2^ = 0.04	*p* = 0.841	*χ* ^2^ = 0.20	*p* = 0.655	*χ* ^2^ = 0.65	*p* = 0.421	*χ* ^2^ = 0.01	*p* = 0.949
Diagnosed with hepatitis	Yes	15 (55.6)	12 (44.4)	19 (70.4)	8 (29.6)	21 (77.8)	6 (22.2)	23 (85.2)	4 (14.8)
	No	318 (50.1)	317 (49.9)	330 (52.0)	305 (48.0)	342 (53.9)	293 (46.1)	491 (77.3)	144 (22.7)
		*χ* ^2^ = 0.31	*p* = 0.58	*χ* ^2^ = 3.518	*p* = 0.061	*χ* ^2^ = 5.98	*p* = 0.014	*χ* ^2^ = 0.92	*p* = 0.337
Vaccination status	Yes	166 (65.9)	86 (34.1)	157 (62.3)	95 (37.7)	161 (63.9)	19 (36.1)	226 (89.7)	26 (10.3)
	No	167 (40.7)	243 (59.3)	192 (46.2)	218 (53.2)	202 (49.3)	208 (50.7)	288 (70.2)	122 (29.8)
		*χ* ^2^ = 39.46	*p* = < 0.001	*χ* ^2^ = 14.99	*p* = < 0.001	*χ* ^2^ = 13.47	*p* < 0.001	*χ* ^2^ = 33.97	*p* < 0.001
Doses received	0	125 (40.2)	186 (59.8)	148 (47.6)	163 (52.4)	20 (64.5)	11 (35.5)	26 (83.9)	5 (16.1)
	1	19 (61.3)	12 (38.7)	18 (58.1)	13 (41.9)	32 (76.2)	10 (23.8)	39 (92.9)	3 (7.1)
	2	29 (69.0)	13 (31.0)	26 (61.9)	16 (38.1)	68 (71.6)	27 (28.4)	87 (91.6)	8 (8.4)
	3	72 (75.8)	23 (24.2)	65 (68.4)	30 (31.6)	157 (50.5)	154 (49.5)	220 (70.7)	91 (29.3)
	Do not know	88 (48.1)	95 (51.9)	92 (50.3)	91 (49.7)	86 (47.0)	97 (53.0)	142 (77.6)	41 (22.4)
		*χ* ^2^ = 45.16	*p* = < 0.001	*χ* ^2^ = 14.90	*p* = 0.005	*χ* ^2^ = 26.58	*p* < 0.001	*χ* ^2^ = 25.46	*p* < 0.001

^a^Likelihood ratio was used where *χ* ^2^ and Fisher's exact test were not applicable, *f* frequency, *%* percentage, *N* total number of respondents, *n* number of respondents in the sub-group, *HB* hepatitis B, *HC* hepatitis C

## Discussion

### Main findings

The aim of this study was to assess the levels of knowledge, attitude and practice related to hepatitis B and C among international students of UPM and its associated factors. The findings indicated that the percentages of international students in UPM with better knowledge of hepatitis B and C were found to be low. The prevalence of positive attitude among the respondents is relatively low with a little more than half of the students having positive attitude towards the disease. However, the proportion of participants with safer practices towards hepatitis B and C is shown to be good as almost 80 % of the participants exhibited safe practices.

Moreover, correlations were found between combinations of levels of knowledge of hepatitis B and C, attitude and practice scores. Likewise, some factors were found to be associated with knowledge, attitude and practice related to hepatitis B and C.

### Comparison with other studies

The percentages of international students in UPM with better knowledge of hepatitis B and C were found to be low, although is better than results from study among university students in Karachi, which showed the level of knowledge of hepatitis B and C combined to be 39 % [14]. This may be explained by the mandatory hepatitis test for all prospective students to be enrolled here in UPM. The prevalence of positive attitude among the respondents is 54.8 %, indicating that more than half of the respondents have positive attitude towards the disease. This is similar to the findings of Razi et al. [16]. The positive attitude towards the diseases by the students could be explained by the on-going HIV campaign globally. The proportion of participants with safer practices towards hepatitis B and C is shown to be high. This is similar to the findings of Razi et al. [16] too. This finding could also be explained by popular HIV campaign on-going globally as well.

Females represented a significantly higher proportion of respondents with better knowledge of hepatitis C as compared with males which is similar to the findings of Khan et al. [14]. For hepatitis B, there is no significant association found which is similar to findings of a previous study [17]. However, it contradicts findings of Roya Mansour-Ghanae and co-researchers who found females to have better knowledge of hepatitis B [18].

The faculty of the participants in this study was significantly associated with hepatitis B knowledge as respondents from the faculty of sciences showed better knowledge of hepatitis B compared to those in faculties of arts and humanities. This is as reported previously in a study among university students in Pakistan [16]. This could be explained by the fact that respondents from the faculty of sciences are more likely to get curricular knowledge of the disease along their study unlike respondents from faculties related to arts and humanities.

Nonetheless, study level was also associated with the level of knowledge of hepatitis B as PhD students showed better knowledge compared to masters and undergraduate students. This result corroborates what was reported in previous study [19]. The finding can be supported by the fact that the more student advances in his/her study the more likely the student is to know more about his/her study area and other related knowledge and skills which can be obtained through seminars, journal clubs among other media.

There were significant association between the knowledge of hepatitis B and C and the family history in relation to hepatitis. Respondents with family members or relatives diagnosed with hepatitis showed better knowledge compared to respondents with no family history of hepatitis. Likewise, respondents with friend diagnosed with hepatitis significantly showed better knowledge of hepatitis compared to those who do not have. This result corresponds with the results of previous study that indicated that having a family member or a friend diagnosed with hepatitis is a predictor of hepatitis B knowledge [20]. However, the result goes contrary to the findings of Darwish and Khaldi’s study [21].

Likewise vaccination status and number of hepatitis vaccine doses received were significantly associated with knowledge of hepatitis B and C. This contradicts results of Darwish and Khaldi who found no significant association between vaccination status of medical students and knowledge of hepatitis B [21]. This may not be surprising as the effect of vaccination may be neutralized by the active knowledge of the disease given to the students during their medical training.

The age of the respondents in this study was significantly associated with positive attitude towards hepatitis B and C. This is in conformity with the findings of a previous study conducted among medical students [14].

Faculty of the respondents in this study was significantly associated with better attitude towards hepatitis B and C. Respondents in the faculty of sciences showed better attitude as compared to those in faculties of arts and humanities. This is as reported previously in a study among university students in Pakistan [16]. Moreover, study level is also associated with positive attitude towards hepatitis B and C. The PhD students among the respondents showed better attitude compared to masters and undergraduate students. This is in keeping with what was reported among students of Guilan University Rast Iran [18]. Moreover, the nationality of the participants was significantly associated with their attitude towards hepatitis B and C with Africans having better attitude compared to other nationalities.

Marital status of the respondents in this study was shown to be associated with the attitude of the respondent towards hepatitis B and C. Ever married respondents had better attitude compared to singles. This contradicts findings of previous study that reported marital status having no significant influence on attitudes of medical students towards hepatitis B and C [18]. As mentioned earlier, this could be a result of formal knowledge of the disease that the medical students received during their medical training as well as their interaction with hepatitis patients. Most of the respondents in this study lack this type of privileged information.

There was significant association between attitude towards hepatitis B and C with family history of hepatitis. Respondents with relatives diagnosed with hepatitis showed better attitude towards the disease compared to those who do not have family history of hepatitis. Likewise, respondents with friends diagnosed with hepatitis significantly showed better knowledge of hepatitis compared to those who do not have. This finding contradicts what was obtained in a previous study [18]. The participants in this study that were diagnosed with hepatitis in the past, or were vaccinated against hepatitis B had higher proportions of those with better attitude towards the disease as compared with those who do not. This is determined with the number of doses of the vaccine received directly related to the attitude of the respondents. However, this was contrary to the findings of Mansour-Ghanaei et al. [18]. All the differences between this study and Mansour-Ghanaei’s study could be explained by the difference in study population.

Gender was significantly associated with safer practices towards hepatitis B and C. Females significantly had higher proportions of those with safer practices towards the disease compared to males. This could be explained by the fact that females generally could be more cautious in their day-to-day life routines compared to males. Nonetheless, this finding is similar to what is obtainable in a previous study [22].

The age of the participants in this study was associated with safer practices towards hepatitis B and C. Participants aged 30 years and above had higher proportion of those with better knowledge compared to other younger age groups. This may be explained by the fact that age of the respondents is linked to their study level with the younger age group being undergraduates and the elderly being PhD students. This is contrary to the findings of another study which demonstrated no significant contribution of age to safer practices towards hepatitis B and C [22].

Marital status of the respondents in this study was shown to be associated with safer practices towards hepatitis B and C of the respondents. The ever married respondents had higher proportion of those with safer practices compared to single respondents. This could be explained by the fact that married individuals are more likely to be subjected to mandatory test during ante natal care. These mandatory tests that may include HIV and hepatitis may prompt the couple to become better informed about the diseases they were investigated for, and to have acquired knowledge that could shape their practices towards it. However, other factors not captured or thought of in this study could explain the finding.

Study level of the respondents was significantly associated with practices of the respondents towards hepatitis B and C. The proportion of those with safer practices increased with the increasing level of study as the undergraduates had the least proportion, while the PhD students had the highest proportion. The finding may be explained by the fact that the more a student advances in his/her study the more likely the student is to know more about his/her study area and other related knowledge and skills, which can be obtained through seminars, journal clubs among other media. As a result, the knowledge obtained may change the practices of that individual positively. This is similar to the finding of a previous study that showed that educational level is significantly associated with attitude towards hepatitis B [22].

There were significant associations between practices towards hepatitis B and C with vaccination status and doses of hepatitis B vaccine received. Respondents vaccinated against hepatitis B had safer practices as compared to those that have not been vaccinated. Likewise, those who received 2 or 3 doses of the vaccine had safer practices compared to those who received only one dose. This can be explained by the fact that those that received complete hepatitis B vaccine may be more conscious health wise and be ready to do anything possible to safeguard their health.

## Conclusion

It can be concluded that the levels of knowledge of hepatitis B and C among the respondents as well as attitude towards the disease were marginally low, although, the practices towards the disease were reasonably good. The study level, faculty, age, nationality, marital status and gender of the respondents were significantly associated with their levels of knowledge, attitude and practices towards hepatitis B and C.

The significance of the study include that: it would add to the body literature as there are very scanty scholarly write-ups on hepatitis-related knowledge, attitude and practice in Malaysia and among international students globally. This paucity has exposed the gap in hepatitis-related knowledge and attitude among international students of UPM that needed to be covered. Finally, it will serve as basis for research including intervention studies regarding hepatitis B and C knowledge, attitude and practices among international students worldwide.

Although, the study was limited by being cross-sectional study, efforts were made to design the study such that the respondents may serve as much as the representatives of the study population as possible. This was achieved by employing probability sampling and reaching out to reasonable number of respondents in a short period of time to avoid any contamination. Similarly, the response rate was reasonable and the study instrument was validated prior to use.
